# Comparing the accuracy of Demirjian and Nolla methods and establishing a new method for dental age estimation in northeastern Chinese children

**DOI:** 10.1080/20961790.2021.2024655

**Published:** 2023-02-12

**Authors:** Dan Wen, Zhiyu Ding, Zhikai Tian, Wenshuang Wu, Weifeng Qu, Wei He, Chudong Wang, Jienan Li, Lagabaiyila Zha, Ying Liu

**Affiliations:** a Department of Forensic Medicine, School of Basic Medical Sciences, Central South University, Changsha, China; b Department of Orthopaedics, The Third Xiangya Hospital of Central South University, Changsha, China; c Department of Oral Implantology, Xiangya Stomatological Hospital, Central South University, Changsha, China; d Clinical Laboratory of Xiangya Stomatological Hospital, Central South University, Changsha, China

**Keywords:** Forensic science, forensic odontology, dental age estimation, Demirjian method, Nolla method, northeastern Chinese children

## Abstract

Dental age estimation plays an important role in the field of clinic medicine and forensic medicine. The Demirjian and Nolla methods are common scoring methods for dental age estimation but there was no research about the comparison of accuracy of these two methods in northeastern Chinese children. Hence, in this study, we compared the accuracy of these two methods to explore more suitable method for our studied population. We collected 535 orthopantomograms from northern Chinese children aged from 6 to 15 years and divided them into training dataset and testing dataset according to the ratio of 7:3. The dental age of training dataset were estimated using Demirjian and Nolla methods, respectively. The results suggested that the mean differences of these two methods were 0.24 and −0.40 years, and mean absolute difference were 0.65 and 0.59 years. Then to further improve the accuracy of dental age assessment, the new improved formulas and dental age conversion tables were established after analyzing the relationship between the sum scores based on Nolla method and chronology age in training dataset. According to the new method used in testing dataset, the minimum value of mean difference (0.00) and mean absolute difference (0.49) were obtained, which are largely smaller than that of Demirjian and Nolla methods. The new developed method and dental age conversion scales may be more suitable dental age estimation method for northeastern Chinese children.

## Key points

We collected 535 orthopantomograms from northern Chinese children aged from 6 to 15 years and divided them into training dataset and testing dataset according to the ratio of 7:3.

The mean differences of Demirjian and Nolla methods in training dataset were 0.24 and −0.40 years, and mean absolute difference were 0.65 and 0.59 years.

The new improved formulas and dental age conversion tables were established after analyzing the relationship between the sum score based on Nolla method and chronology age in training dataset.

According to the new method used in testing dataset, the minimum value of mean difference (0.00) and mean absolute difference (0.49) were obtained, which are largely smaller than that of Demirjian and Nolla methods.

## Introduction

Age estimation plays a critical role in a lot of disciplines including forensic science, anthropology, pediatric dentistry, orthodontics and so on [1]. For forensic science and anthropology, age estimation is needed to solve some criminal and civil cases and identify the victims of mass disasters [2–4]. For pediatric dentistry and orthodontics, defining clinical diagnosis and determining the time of treatment also needs the assistance of age estimation [5, 6]. There are several methods to estimate chronological age based on the human growth and development including those on the basis of the development of teeth, skeleton, stature, etc. [7]. Due to some special advantages, dental age estimation has drawn more and more attention from research scholars. The development of teeth has a close relation with the human growth, but it is more affected by genes than by endocrinopathy and malnutrition compared with other methods of age estimation [8]. Moreover, teeth are the most indestructible organ in human body and can remain unchanged a long of time after death [9]. Dental age estimation is applied in not only living persons but also cadavers. The most common method to observe the development of teeth is radiography, which is convenient, economical, and non-invasive [10].

The history of dental age estimation can be track back to ancient Roma. They determined whether adolescents were suitable for service according to the complete eruption of the second molars [11]. After the X-ray technology invented by Roentgen, dental age estimation based on radiography has been gradually accepted [12]. In the last century, the development of teeth was widely used to assess the age of child labour [13]. If child labour was younger than 9 years, factories were not allowed to employ them. Moreover, child labour would be restricted from working hours if they were between the ages of 9 and 12 years [13]. With the progress of radiography and the increase of actual demands, many different methods of dental age estimation have been gradually reported [5, 6, 14–20]. They are on the basis of the eruption and the formation of teeth. Compared with the eruption of teeth, the formation of crowns and roots is less affected by the environmental factors and can provide more precise data [21]. The formation of teeth is mainly divided into three stages: beginning calcification, crown completion and root completion [21]. Meanwhile, they fall into two broad categories: atlas methods and scoring methods [22]. The Schour’s and Masseler’s method belong to the atlas methods [16]. There are two common scoring methods: Demirjian and Nolla methods [5, 18]. Due to the accuracy and simplicity, the scoring methods have been investigated by more and more scholars in recent years.

The Nolla’s laboratory came up with the Nolla method according to the investigation of the development of teeth of 3 402 Michigan children in 1952 [18]. There were 10 stages (1–10) for the development of each tooth after researching the permanent teeth. Then, the Demirjian method was reported by Demirjian’s laboratory in 1973, which was based on the study of 2 928 French Canadian children [5]. The development of teeth was divided into eight stages (A–H).

In Chinese children, the results of dental age estimation using Demirjian and/or Nolla methods for different research were different. Only Han et al. [23] reported that the chronological age was overestimated using Nolla method for northern Chinese, and the mean absolute difference between dental age and chronological age reached 0.72 years [23]. For Demirjian method, the overestimation of chronological age was found in part of northern and western Chinese, but the underestimation was shown in part of central southern and northern Chinese [24–27]. However, the accuracy of Demirjian and Nolla methods has not been compared in northeastern Chinese. When the two methods are inappropriate, a new improved method for dental age estimation also needs to be established.

Hence, in this study, we collected 535 orthopantomograms (OPGs) from northeastern Chinese children aged from 6 to 15 years and estimated dental age using Demirjian and Nolla methods, respectively. The aim of this study is to compare the accuracy of Demirjian and Nolla methods in dental age estimation of northeastern Chinese children. If necessary, we will explore a new improved method for our studied population based on above methods.

## Materials and methods

### Collection of samples

A retrospective cross-sectional study was conducted in a sample of 535 OPGs of healthy northern Chinese children (269 males and 266 females) between the ages of 6 and 15 years. The samples were divided into training dataset (180 males and 178 females) and testing dataset (89 males and 88 females) according to the ratio of 7:3. The sample is roughly evenly distributed across all age groups and genders. All radiographs were collected from the Department of Dentistry, Chifeng Municipal Hospital, Inner Mongolia Autonomous Region, China between 2014 and 2019 with institutional approval. The writing informed consent was signed by the parents or guardians of the children. Moreover, the Ethics Committee of Xiangya Hospital approved this study.

### Selection criteria

The children with the clear and complete radiographs, who were living in the Inner Mongolia Autonomous Region and aged from 6 to 15 years, were included. The detailed and accurate dental records were also needed to verify the data of birth, gender and data of radiography of children. However, we also needed to exclude any radiographs with incomplete image or image distortion due to the incorrect behaviours during exposure. Moreover, the children with any disease (syndrome, oral cleft, tooth agenesis and supernumerary teeth), trauma or malnutrition that could affected the development of teeth should also be excluded.

### Calculation of chronological age (CA)

The numerical ID, gender, date of birth and date of radiography were recorded in an Excel table and then the CA was obtained by calculating the difference between the date of birth and the date of radiography. The age and sex distributions were shown in the Table 1.

**Table 1.** Age and sex distribution of training dataset and testing dataset from the study population.

Age groups (year)	Training dataset		Testing dataset		Total
	Males	Females	Males	Females	
6.01–7.00	20	18	9	8	55
7.01–8.00	20	20	10	10	60
8.01–9.00	20	20	10	10	60
9.01–10.00	20	20	10	10	60
10.01–11.00	20	20	10	10	60
11.01–12.00	20	20	10	10	60
12.01–13.00	20	20	10	10	60
13.01–14.00	20	20	10	10	60
14.01–15.00	20	20	10	10	60
Total	180	178	89	88	535

### Estimation of dental age (DA)

All exported images were gathered in a computer folder after converting the OPGs into the jpg format. The assessment of images was conducted by two different researchers using the Adobe Photoshop 7 software (San Jose, CA, USA). To avoid errors, only the numerical ID would be provided to them, not the gender, date of birth and date of radiography. Then the DA would be obtained using the Demirjian and Nolla methods described in the corresponding published reports, respectively [5, 18].

### Statistical analysis

The Kappa statistic was used to evaluate the inter-observer and intra-observer reproducibility. Two observers assessed the same 50 radiographs using the two methods without the information of their respective assessments to analyze the inter-observer reproducibility. Similarly, one observer re-examined the 50 radiographs using the two methods without the information of the first examination after a period of 4 weeks to analyze the intra-observer reproducibility.

The mean CA and mean DA of Demirjian and Nolla methods for both genders and all age groups were calculated to analyze the age and gender distribution in the training dataset. The mean differences (MD) and mean absolute differences (MAD) between DA and CA of these two methods for both genders and all age groups were also analyzed to compare the accuracy of the Demirjain and Nolla methods in the training dataset. The value of MD greater than 0 meant an overestimation of CA while the value of MD less than 0 indicated an underestimation of CA. The Kolmogorov-Smimov test was applied to analyze the normality of distribution of samples. For analyzing the significant difference between DA and CA of a normal distributed samples, the paired *t*-test was applied. The Wilcoxon signed rank test was used to analyze the significant difference between DA and CA of a non-normal distributed samples. The threshold of *P*-value of significant difference was 0.05. These statistical analyses were conducted with the SPSS version 20.0 software (Armonk, NY, USA).

### Establishment of new method

The relationship between stage sum score and CA was identified in the training dataset. Then a best appropriate formula was developed based on above relationship. Finally, a new adapted dental age conversion table for dental age estimation in northeastern Chinese children was established. The CA, DA, MD and MAD of Demirjian, Nolla and new adapted methods for both genders and all age groups were also assessed to compare the accuracy for dental age estimation in the testing dataset.

## Results

### Analysis of reproducibility

The Kappa values of intra-observer agreement for Demirjian and Nolla methods were 0.962 and 0.954, respectively. While the values of inter-observer agreement for Demirjian and Nolla methods were 0.957 and 0.944 using Kappa statistic.

### Accuracy of Demirjian method

The mean CA, mean DA, MD and MAD for males, females and total samples for Demirjian method based on training dataset were summarized in Table 2. For total samples, the mean CA and DA were (10.60 ± 2.43) years and (10.83 ± 2.59) years respectively. The MD and MAD for total samples using Demirjian method were 0.24 and 0.65 years, which all showed statistically significant differences. For males, the mean CA was (10.56 ± 2.48) years, and the mean DA was (10.73 ± 2.60) years. The MD and MAD of males using the Demirjian method were 0.18 and 0.65 years, respectively. Except that the CA of age group 8.01–9.00, 9.01–10.00 and 13.01–14.00 of males were underestimated, the CA of other age groups were all overestimated. The MD of age groups 6.01–7.00 and 14.01–15.00 of males showed statistically significant differences. For females, the mean CA was (10.64 ± 2.40) years. The DA of females using Demirjian method was (10.94 ± 2.59) years with MD of 0.30 and MAD of 0.66. Except that the CA of age group 8.01–9.00 of females were underestimated, the CA of other age groups were all overestimated. The MD of age group 7.01–8.00, 12.01–13.00 and 14.01–15.00 of females showed no statistically significant differences.

**Table 2.** The mean CA, mean DA, MD and MAD of both sexes and all age groups for Demirjian method based on training dataset.

	Age group	CA(SD)	DA(SD)	MD(SD)	*P-*value	95%CI of MD	MAD(SD)
Males	6.01–7.00	6.74 (0.34)	7.40 (0.25)	0.66 (0.35)	0.00*	0.50–0.83	0.66 (0.35)
	7.01–8.00	7.54 (0.27)	7.67 (0.37)	0.13 (0.51)	0.85	(−0.11)−0.37	0.34 (0.40)
	8.01–9.00	8.70 (0.34)	8.60 (1.04)	−0.10 (1.00)	0.67	(−0.56)−0.37	0.74 (0.66)
	9.01–10.00	9.68 (0.31)	9.34 (1.26)	−0.35 (1.22)	0.22	(−0.92)−0.22	1.08 (0.62)
	10.01–11.00	10.82 (0.26)	11.13 (0.72)	0.32 (0.83)	0.07	(−0.07)−0.71	0.73 (0.47)
	11.01–12.00	11.51 (0.34)	11.73 (0.52)	0.23 (0.52)	0.06	(−0.01)−0.47	0.41 (0.38)
	12.01–13.00	12.36 (0.26)	12.55 (0.53)	0.19 (0.58)	0.09	(−0.08)−0.46	0.51 (0.31)
	13.01–14.00	13.29 (0.26)	13.22 (0.83)	−0.08 (0.82)	0.67	(−0.46)−0.31	0.64 (0.50)
	14.01–15.00	14.37 (0.26)	14.96 (0.72)	0.59 (0.70)	0.00*	0.26–0.91	0.73 (0.55)
Total males		10.56 (2.48)	10.73 (2.60)	0.18 (0.81)	0.00*	0.06–0.30	0.65 (0.52)
Females	6.01–7.00	6.76 (0.29)	7.17 (0.23)	0.41 (0.28)	0.00*	0.27–0.55	0.42 (0.27)
	7.01–8.00	7.76 (0.32)	7.85 (0.48)	0.09 (0.57)	0.51	(−0.18)−0.35	0.49 (0.28)
	8.01–9.00	8.87 (0.24)	8.21 (0.78)	−0.65 (0.82)	0.00*	(−1.04)–(−0.27)	0.89 (0.53)
	9.01–10.00	9.75 (0.30)	10.42 (1.38)	0.66 (1.30)	0.03*	0.06–1.27	1.04 (1.00)
	10.01–11.00	10.72 (0.32)	11.64 (0.98)	0.92 (0.99)	0.00*	0.45–1.38	0.94 (0.98)
	11.01–12.00	11.49 (0.25)	11.88 (0.34)	0.39 (0.41)	0.00*	0.20–0.58	0.47 (0.31)
	12.01–13.00	12.30 (0.15)	12.74 (0.82)	0.44 (0.78)	0.13	0.08–0.81	0.64 (0.62)
	13.01–14.00	13.36 (0.29)	13.83 (0.70)	0.47 (0.70)	0.01*	0.15–0.80	0.68 (0.49)
	14.01–15.00	14.32 (0.29)	14.33 (0.42)	0.01 (0.37)	0.92	(−0.16)−0.18	0.33 (0.14)
Total females		10.64 (2.40)	10.94 (2.59)	0.30 (0.86)	0.00*	0.18–0.43	0.66 (0.63)
Total samples	6.01–15.00	10.60 (2.43)	10.83 (2.59)	0.24 (0.84)	0.00*	0.15–0.33	0.65 (0.57)

CA: chronological age; DA: dental age; MD: mean differences (DA–CA); MAD: mean absolute differences; SD: standard deviation; CI: confidence Interval.

*P-*value: obtained using paired samples *t*-test or Wilcoxon signed rank test.

* Values that showed significant difference.

### Accuracy of Nolla method

The mean CA, mean DA, MD and MAD for males, females and total samples for Nolla method based on training dataset were summarized in Table 3. For total samples, the mean CA and DA were (10.60 ± 2.43) years and (10.19 ± 2.47) years, respectively. The MD and MAD for total samples using Nolla method were −0.40 and 0.59 years, which all showed statistically significant differences. For males, the mean CA was (10.56 ± 2.48) years, and the mean DA was (10.16 ± 2.41) years. The MD and MAD of males using the Nolla method were −0.40 and 0.56 years, respectively. Except that the CA of age group 6.01–7.00 of males were overestimated, the CA of other age groups all were underestimated. The MD of age groups 7.01–8.00, 8.01–9.00, 9.01–10.00, 10.01–11.00, 12.01–13.00, 13.01–14.00 and 14.01–15.00 of males showed statistically significant differences. For females, the mean CA was (10.64 ± 2.40) years. The DA of females using Nolla method was (10.23 ± 2.54) years with MD of −0.40 and MAD of 0.62. The CA of age groups were all underestimated. The MD of age groups 6.01–7.00, 7.01–8.00, 8.01–9.00, 11.01–12.00, 12.01–13.00, 13.01–14.00 and 14.01–15.00 of females showed statistically significant differences.

**Table 3.** The mean CA, mean DA, MD and MAD of both sexes and all age groups for Nolla method based on training dataset.

	Age group	CA(SD)	DA(SD)	MD(SD)	*P-*value	95%CI of MD	MAD(SD)
Males	6.01–7.00	6.74 (0.34)	6.81 (0.52)	0.07 (0.42)	0.44	(−0.12)–0.27	0.36 (0.21)
	7.01–8.00	7.54 (0.27)	7.22 (0.44)	−0.31 (0.45)	0.01*	(−0.53)–(−0.10)	0.44 (0.32)
	8.01–9.00	8.70 (0.34)	8.17 (0.64)	−0.53 (0.57)	0.00*	(−0.79)–(−0.26)	0.57 (0.53)
	9.01–10.00	9.68 (0.31)	9.21 (0.85)	−0.47 (0.88)	0.03*	(−0.89)–(−0.06)	0.81 (0.58)
	10.01–11.00	10.81 (0.26)	10.42 (0.77)	−0.40 (0.79)	0.04*	(−0.77)–(−0.02)	0.68 (0.56)
	11.01–12.00	11.51 (0.34)	11.32 (0.48)	−0.19 (0.44)	0.05	(−0.40)–0.02	0.36 (0.31)
	12.01–13.00	12.36 (0.29)	11.71 (0.37)	−0.65 (0.36)	0.00*	(−0.82)–(−0.48)	0.66 (0.35)
	13.01–14.00	13.29 (0.26)	12.59 (0.62)	−0.70 (0.62)	0.00*	(−0.99)–( −0.41)	0.71 (0.61)
	14.01–15.00	14.37 (0.26)	13.97 (0.49)	−0.40 (0.40)	0.00*	(−0.59)–(−0.21)	0.48 (0.30)
Total males		10.56 (2.48)	10.16 (2.41)	−0.40 (0.61)	0.00*	(−0.49)–(−0.31)	0.56 (0.46)
Females	6.01–7.00	6.76 (0.29)	6.31 (0.41)	−0.44 (0.38)	0.00*	(−0.63)–(−0.25)	0.46 (0.35)
	7.01–8.00	7.76 (0.32)	7.34 (0.68)	−0.42 (0.73)	0.02*	(−0.76)–(−0.07)	0.70 (0.46)
	8.01–9.00	8.87 (0.24)	7.87 (0.83)	−1.00 (0.85)	0.00*	(−1.40)–(−0.60)	1.11 (0.69)
	9.01–10.00	9.75 (0.30)	9.66 (0.87)	−0.09 (0.79)	0.15	(−0.46)–0.28	0.58 (0.54)
	10.01–11.00	10.72 (0.32)	10.62 (0.89)	−0.10 (0.79)	0.16	(−0.47)–0.27	0.59 (0.52)
	11.01–12.00	11.49 (0.25)	11.04 (0.39)	−0.44 (0.45)	0.00*	(−0.65)–(−0.24)	0.48 (0.41)
	12.01–13.00	12.30 (0.15)	11.91 (0.80)	−0.39 (0.74)	0.03*	(−0.74)–(−0.05)	0.72 (0.40)
	13.01–14.00	13.36 (0.29)	12.89 (0.56)	−0.47 (0.59)	0.00*	(−0.75)–(−0.20)	0.56 (0.50)
	14.01–15.00	14.32 (0.29)	14.04 (0.57)	−0.28 (0.45)	0.01*	(−0.49)–(−0.07)	0.39 (0.35)
Total females		10.64 (2.40)	10.23 (2.54)	−0.40 (0.70)	0.00*	(−0.51)–(−0.30)	0.62 (0.51)
Total samples	6.01–15.00	10.60 (2.43)	10.19 (2.47)	−0.40 (0.65)	0.00*	(−0.47)–(−0.33)	0.59 (0.49)

CA: chronological age; DA: dental age; DA: dental age; MD: mean differences (DA−CA); MAD: mean absolute differences; SD: standard deviation; CI: confidence Interval.

*P-*value: obtained using paired samples t-test or Wilcoxon signed rank test.

* Values that showed significant difference.

### Comparison of accuracy of Demrjian and Nolla methods

The Demirjian method overestimated the CA of northeastern Chinese children, while the Nolla method underestimated. The MD for total samples obtained by Demirjian method was smaller than that obtained by Nolla method, but the results of MAD for total samples using these two methods were similar and all larger than 0.5 years. The comparison of MAD was made for all age groups in both males and females using Demirjian and Nolla methods based on training dataset. For age group 6.01–7.00, 8.01–9.00, 9.01–10.00, 10.01–11.00, 11.01–12.00, and 14.01–15.00 of males, the results of MAD using Nolla method were smaller than that using Demirjian method. Especially for age group 9.01–10.00 of males, the values of MAD obtained by Demirjian method were greatly large (>1.0 year). For age group 9.01–10.00, 10.01–11.00, and 13.01–14.00 of females, the results of MAD using Nolla method were smaller than that using Demirjian method. Especially for age group 9.01–10.00 of females, the values of MAD obtained by Demirjian method were larger than 1 year. These results indicated that the Demirjian and Nolla methods were similar for age estimation in northeastern Chinese children, but the Demirjian method had a smaller level of MD and the Nolla method had a smaller level of MAD.

### Establishment of a new improved method

To further improve the accuracy of dental age assessment, the relationship between sum scores based on Demirjian or Nolla method and CA was analyzed in the training dataset (Supplementary Table S1). The values of *R*^2^ of Quandratic function was largest for curve estimation based on these two methods. For both males and females, the largest values of *R*^2^ of Quandratic function was obtained by Nolla method (0.931 and 0.921, respectively). The sum scores based on Nolla method was closely relative with CA with a curve distribution for both males and females. Then the new formulas that were most suitable for males and females were developed based on the above results. The values of *R*^2^ of the new formula were 0.931 for males and 0.921 for females, both showing a strong correlation between sum score and CA. The detailed new formulas for males and females were as follow:

$$\begin{array}{c} \text{Males}:\;\text{Score}=4.916+7.783*\text{CA}-0.232*{\text{CA}}^{2}\\ \text{Females}:\;\text{Score}=-2.751+9.785*\text{CA}-0.331*{\text{CA}}^{2} \end{array}$$

**Figure 1.**
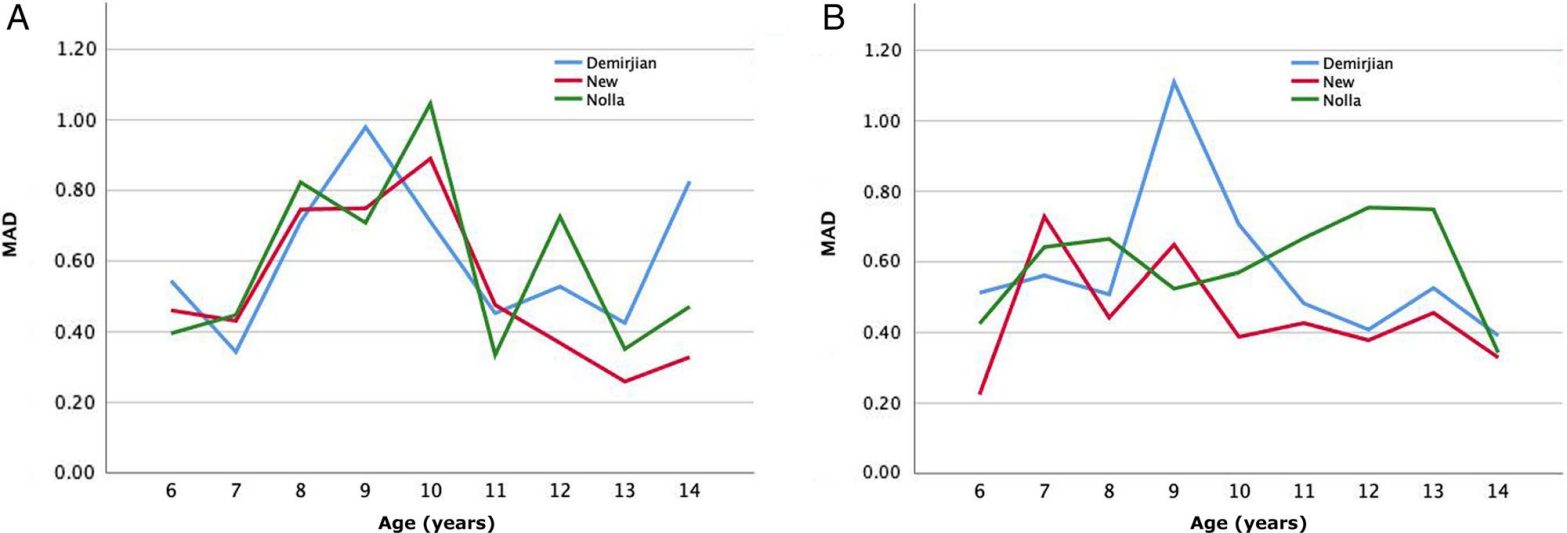
The comparison of mean absolute differences (MAD) for all age groups of males (A) and females (B) in testing dataset using Demirjian, Nolla and new adapted methods.

Then, the new dental age conversion tables for males and females were obtained using above formulas and shown Supplementary Tables S2 and S3. The sum score for females was larger than for males in the same age group, indicating that the teeth development of females was earlier than males. The mean CA, mean DA, MD and MAD for males, females, and total samples for Demirjian, Nolla and new method based on the testing dataset were summarized in Table 4. For total samples, the MD and MAD for total samples using new method were 0.00 and 0.49, which showed no significant differences and were greatly smaller than that of Demirjian and Nolla methods. The comparison of MAD for all age groups in males and females using Demirjian, Nolla and new model methods based on the testing dataset was shown in Figure 1. For males, the MAD of age groups 12.01–13.00, 13.01–14.00 and 14.01–15.00 was largely smaller than that of Demirjian and Nolla methods. For females, except for age groups 7.01–8.00 and 9.01–10.00, the MAD of other seven age groups were largely smaller than that of Demirjian and Nolla methods.

**Table 4.** The mean CA, mean DA, MD and MAD for males, females and total samples for Demirjian, Nolla and new method based on testing dataset.

Method		Males	Females	Total
Demirjian	CA (SD)	10.59 (2.48)	10.65 (2.40)	10.62 (2.44)
	DA (SD)	10.76 (2.66)	10.91 (2.45)	10.83 (2.55)
	MD (SD)	0.17 (0.76)	0.26 (0.68)	0.21 (0.72)
	MAD	0.61 (0.47)	0.58 (0.44)	0.60 (0.45)
Nolla	CA (SD)	10.59 (2.48)	10.65 (2.40)	10.62 (2.44)
	DA (SD)	10.17 (2.54)	10.20 (2.47)	10.18 (2.50)
	MD (SD)	−0.42 (0.66)	−0.46 (0.63)	−0.44 (0.64)
	MAD	0.59 (0.51)	0.60 (0.50)	0.59 (0.50)
New method	CA (SD)	10.59 (2.48)	10.65 (2.40)	10.62 (2.44)
	DA (SD)	10.60 (2.68)	10.64 (2.37)	10.62 (2.52)
	MD (SD)	0.01 (0.69)	−0.01 (0.59)	0.00 (0.64)
	MAD	0.52 (0.45)	0.45 (0.37)	0.49 (0.41)

CA: chronological age; DA: dental age; MD: mean differences (DA−CA); MAD: mean absolute differences; SD: standard deviation.

## Discussion

Dental age estimation plays an important role in the field of clinic medicine and forensic medicine due to some specific advantages of teeth including the most indestructible organ in human body, a close relation with the human growth, and less affection by environmental factors [1–6, 8, 9]. There are many methods to estimate dental age, and the Demirjian and Nolla methods are common scoring methods [5, 18]. In this study, we collected 535 OPGs of healthy northern Chinese children aged from 6 to 15 years, and then compared the accuracy of dental age estimation of the Demirjian and Nolla methods in the studied population.

Now, the Demirjian method is the most popular method and has been studied in multiple populations and regions. According to the previously reported studies, there are two main studied populations Asian and Caucasian [28]. For males, the MD of Asian and Caucasian were 0.28 and 0.38 years using Demirjian method. The MD of females were 0.24 years for Asian but 0.52 years for Caucasian using Demirjian method. Moreover, the studies between 1973 and 2011 based on the French-Canadian dataset showed that the Demirjian method overestimated CA in most populations and regions but only the CA of Venezuelan and western Chinese males were underestimated [25, 29, 30]. The MD for males ranged from −0.08 to 3.04 years and for females ranged from −0.10 to 2.82 years. The dental age estimation of western China using Demirjian method was the most accurate (males: −0.08 years, females: 0.15 years), while the MD of southern India was the largest (males: 3.04 years, females: 2.82 years) [25, 31]. In the southern India and Malaysia, the Nolla method overestimated the CA, and the mean differences (DA−CA) were 0.47 and 0.97 years, respectively [32–33]. However, the CA of Spain and Turkey were underestimated 0.21 and 0.54 years [34–35]. There were nine studies about Demirjian method *versus* Nolla method [23, 33, 35–41]. For India, Bangladesh, Malaysia and northern China, the results of Nolla method were more accurate, but the other five studies (Spain, Turkey, Brazil, Croatia and Portugal) showed that the Demirjian method was a more suitable method. The reasons of the deviations between the different populations have not been clear. Some scholars indicated that the size and distribution of the sample, scoring criteria and statistical analyses may cause these deviations [42], but there was evidence suggesting that the variation in dental maturity exists among different populations. Chaillet et al. [14] compared the dental maturity of eight different countries by collecting 9 577 dental panoramic tomograms and found that the inter-ethnic differences exist in three major groups. The differences in the dental maturity among Korean and Swedish population also showed significant [43]. Demirjian also suggested that the accuracy of conversion of maturity scores to dental ages may be affected by the population differences, and population specific standards may provide a higher level of accuracy [5]. For example, Akhil et al. [44] reported that the Indian-specific formula based on Demirjian method was more reliable in dental age estimation of Kanyakumari population. Similarly, Jayaraman et al. [45] also showed that the dental age estimation based on southern Chinese specific reference data can provide more accuracy results for southern Chinese children [45].

The recommendations for the application of the Demirjian method in China are different between a series of published studies. The only study in China on the use of Nolla method showed a large MAD (0.72 years). However, the accuracy of Demirjian and Nolla methods has not been compared in northeastern Chinese, and a new improved method based on Nolla or Demirjian has not been developed. Hence, the accuracy of Demirjian and Nolla methods was compared to find the more suitable method for northeastern Chinese children. Similar to most of the above studies, the Demirjian method overestimated the CA, but the Nolla method underestimated. Overall, the MAD of total samples of these two methods were larger than 0.5 years, showing that these two methods also need to be improved for estimating the dental age of northeastern Chinese children. But the Demirjian method had a smaller level of MD and the Nolla method had a smaller level of MAD. Then the new improved formulas and dental age conversion tables for males and females were established after analyzing the relationship between the sum scores based on Nolla method and CA. According to the new method, the minimum value of MD (0.00) and MAD (0.49) were obtained. The accuracy of dental age estimation has been greatly improved by the new adapted method based on Nolla method. However, the limited number of testing dataset in our study may affect the assessment of the accuracy of this model.

In summary, both the Demirjian and Nolla methods need improvement for estimating the dental age in northeastern Chinese children, but the Demirjian method had a smaller level of MD and the Nolla method had a smaller level of MAD. Most studies of Demirjian and/or Nolla method used in China just focus on comparing the accuracy, not developing a new adapted method. Now we reported the new adapted method based on Nolla method, which could greatly improve the accuracy of age estimation for northeastern Chinese children comparing with the Demirjian and Nolla methods. The new developed method and dental age conversion scales can help find more suitable dental age estimation method for northeastern Chinese children.
